# The trilemma among CO_2_ emissions, energy use, and economic growth in Russia

**DOI:** 10.1038/s41598-023-37251-5

**Published:** 2023-06-23

**Authors:** Cosimo Magazzino, Marco Mele, Carlo Drago, Sevda Kuşkaya, Cesare Pozzi, Umberto Monarca

**Affiliations:** 1 Department of Political Sciences, Roma Tre University, Rome, Italy; 2 “Niccolò Cusano” University, Rome, Italy; 3 Justice Vocational College, Erciyes University, Kayseri, Turkey; 4 Department of Economics, University of Foggia, Foggia, Italy

**Keywords:** Environmental impact, Energy and society, Environmental economics, Environmental sciences, Environmental social sciences

## Abstract

This paper examines the relationship among CO_2_ emissions, energy use, and GDP in Russia using annual data ranging from 1990 to 2020. We first conduct time-series analyses (stationarity, structural breaks, cointegration, and causality tests). Then, we performed some Machine Learning experiments as robustness checks. Both approaches underline a bidirectional causal flow between energy use and CO_2_ emissions; a unidirectional link running from CO_2_ emissions to real GDP; and the predominance of the “neutrality hypothesis” for energy use-GDP nexus. Therefore, energy conservation measures should not adversely affect the economic growth path of the country. In the current geopolitical scenario, relevant policy implications may be derived.

## Introduction

The increase in the production and consumption activities of societies, especially after the industrial revolution, is seen as the main reason for the rise in Greenhouse Gases (GHG) in the atmosphere (EPA^1^). The vast majority of GHG is released as carbon dioxide (CO_2_) emissions as a result of burning fossil fuels (Ardakani and Seyedaliakbar^2^). CO_2_ emissions account for approximately 80% of total GHG emissions (EPA^3^). According to the IPCC^4^, anthropogenic CO_2_ emissions resulting from the combustion of fossil fuels increase global warming and cause climatic deterioration (Alam et al.^5^).

Since CO_2_ emissions depend on the population and changing economic, technological, and social conditions, environmental pollution is considered a by-product of the growth-development process. (Kahouli^6^). Therefore, climate change has been the most challenging environmental problem of our time, and the determination of the environment-energy-economic growth link has attracted the attention of researchers (Acheampong et al.^7^).

It is possible to classify the studies in the existing environment-economics literature as (i) papers investigating the relationship between economic growth-energy consumption (Magazzino^8^^,^^9^; Balsalobre-Lorente and Álvarez-Herranz^10^; Taghvaee et al.^11^; Brady and Magazzino^12^; Ibrahiem^13^; Balcilar et al.^14^; Acheampong et al.^1,7^; Xin-gang and Jin^15^); (ii) studies estimating the nexus between economic growth and environment (Alvarado and Telodo^16^; Nasrollahi et al.^17^; Wang and Lee^18^; Wang et al.^19^); and (iii) papers analyzing the correlation among economic growth-energy consumption-environment (Magazzino^20^; Balsalobre-Lorente et al.^21^; Benali and Feki^22^; Peng and Wu^23^; Hasan et al.^24^; Kongkuah et al.^25^). This study can be defined as complementary to the previous papers in the context of energy economics.

Moreover, the Russian-Ukrainian conflict is one of the factors that has inflamed the already “hot” prices of commodities: not only those used as energy sources (oil and gas) but also industrial metals and agricultural products (wheat and corn) that feed many countries of the world. Already before the conflict, commodity prices had risen sharply, driven by the economic recovery following the Corona Virus Disease 2019 (COVID-19) pandemic. The conflict has then complicated the scenario, even if an important aspect should be highlighted: since the financial markets thrive on expectations, the rising prices of commodities discount in advance future risks.

Thanks to the large dimension of their territory, Russia and Ukraine are major producers of agricultural raw materials. The two countries account for nearly a third of the world’s wheat exports and 15% of maize exports. Russia is also rich in natural resources. In addition to natural gas and oil, it boasts 25% of world palladium exports, 13% of nickel and platinum, and about 3% of aluminum and copper. Moreover, approximately 38% of the natural gas consumed annually in Europe comes from Russia (Magazzino and Mele^26^).

Also, Russia is one of the world’s leading countries in terms of energy infrastructure and economic performance. It is known for its vast reserves of natural resources, including oil, natural gas, coal, and minerals. According to the IEA^27^ report, Russia maintained its position as the world’s leading exporter of natural gas in 2021, while ranking second in crude oil and condensate exports, just behind Saudi Arabia. It was also the third largest coal exporter, behind Indonesia and Australia. At the same time, Russia holds a significant position in the global energy landscape, playing a crucial role as one of the top three crude oil producers behind Saudi Arabia and the United States (US). These powerful fuel reserves are of great importance to the economy of the Russian Federation. For instance, in 2021 approximately 45% of Russia’s federal budget was supported by the revenues derived from oil and gas (IEA^28^).

Russia ranks as the fourth-largest emitter of GHG globally, right after China, the US, and India. Furthermore, it holds the distinction of being the third-highest historical carbon emitter worldwide, contributing approximately 7% to the cumulative global CO_2_ emissions (Carbonbrief^29^). Despite maintaining its fossil fuel industries, the country is gradually increasing its investments in renewable energy and taking steps toward climate initiatives. In 2019, Russia became a signatory of the Paris Agreement which is an international treaty focused on addressing global warming. Hence, in 2020, the Russian government approved a national action plan to adapt to climate change (Statista^30^).

Considering Russia’s significant natural endowments including energy sources and its economic growth and contribution to the GHG, the scope of this study is to estimate the nexus among energy consumption, economic growth, and CO_2_ emissions in Russia for the years 1990–2020. In the analysis, a time-series analysis is performed, followed by Machine Learning (ML) tests on the causality flows and ML clustering analyses. Basically, the sample choice is related to the following motivations: (a) Russia has the largest proven natural gas reserves in the world (Statista^31^); (b) according to the EIA^32^ report, Russia was the world’s third-largest producer of petroleum and other liquids in 2020 after the USA and Saudi Arabia; (c) it is one of the countries with the highest per capita GHG emissions (C2ES^33^).

Furthermore, the current conflict between Russia and Ukraine raises some cautions. Russia is facing a deep financial and economic recession, which puts significant pressure on policymakers to focus on short-term economic recovery measures rather than long-term environmental sustainability goals. The Russian government chose to prioritize economic growth, which has resulted in an increased focus on oil and gas production, a significant contributor to environmental degradation. Additionally, the Russian government has historically been skeptical of environmental policies that could potentially harm the country’s economic interests. The government has favoured a centralized approach to environmental decision-making, which has resulted in a lack of public participation and transparency in environmental policy development. Moreover, the current political climate in Russia does not prioritize environmental policies, and the government’s response to environmental issues has been relatively weak compared to other countries. Moreover, the country has significant reserves of oil and gas, which are essential to its economy. Therefore, the government may not be willing to invest in alternative energy sources, which could potentially weaken its economic interests.

The main contributions of this paper to the literature are threefold. Firstly, the literature on the nexus among economic growth, CO_2_ emissions, and energy consumption for Russia is extremely limited. Moreover, only standard techniques have been performed. Secondly, to the best of our knowledge, no ML experiments have been conducted for the Russian case in the relevant literature. Moreover, we also applied to our dataset some recent time-series tests on stationarity, cointegration, and causality. In this way, two completely different empirical methodologies are shown, giving robust results. Finally, after the conflict between Russia and Ukraine, there was a surge in the prices of natural resources (especially gas and oil). Therefore, analyzing the Russian energy sector takes on even greater relevance today.

The outline of this article is as follows: in Sect. “Literature overview” the relevant literature is presented. The econometric methodology is given in Sect. “Methodology and data”. Section “Empirical findings” consists of the empirical results and their discussions. Finally, Sect. “Conclusions and policy implications” gives conclusions and policy implications.

## Literature overview

Climate change and global warming are among the most important ongoing problems in the world, almost affecting everything adversely from economies (industrial production, agriculture, tourism, service, and other sectors) to demography (urbanization and ruralization), and hence the quality of life in general. Thus, these phenomena should be monitored by the level of CO_2_ emissions. Therefore, investigating the sources and consequences of CO_2_ emissions might be considered one of the most important research topics in the literature.

In this section, the relevant literature is evaluated in three different groups. Since one of the most important factors causing global warming is CO_2_ emissions due to energy use, the first group consists of papers investigating the relationship between energy consumption and CO_2_ emissions. Many seminal types of research have analyzed the nexus between energy consumption and the environment (Soytas and Sari^34^; Magazzino^35^^,^^36^; Zoundi^37^; Bilgili et al.^38^).

According to Table 1, the related research can be classified into, based on their samples, (a) single-country studies (Soytas and Sari^34^; Lin and Moubarak^39^; Jaforullah and King^40^; Ben Jebli and Ben Youssef^41^; Beşer and Beşer^42^; Mirzaei and Bekri^43^; Bekhet and Othman^44^; Sinha and Shahbaz^45^; Waheed et al.^46^; Kuşkaya and Bilgili^47^; Kim et al.^48^; Kuşkaya^49^; Ozgur et al.^50^; Apergis et al.^51^; Kartal et al.^52^; Kuşkaya et al.^53^; Mukhtarov et al.^54^) or (b) multi-country studies (Apergis et al.^55^; Zoundi^37^; Belaïd and Zrelli^56^; Chontanawat^57^; Saidi and Omri^58^; Adebayo et al.^59^). It is seen that auto-regressive distributed lags (ARDL), vector error correction model (VECM), and Granger causality (GC) analyses are conducted mostly in studies for a single country as seen in Table 1. In most of the papers in Table 1, there is a consensus that increasing the usage of renewable energy reduces CO_2_ emissions (Lin and Moubarak^39^; Jaforullah and King^40^; Ben Jebli and Ben Youssef^41^; Mirzaei and Bekri^43^; Bekhet and Othman^44^; Ozgur et al.^50^; Apergis et al.^51^; Kuşkaya et al.^53^; Mukhtarov et al.^54^). On the other hand, according to Beşer and Beşer^42^, total energy usage increased CO_2_ emissions for the Turkish economy from 1960 to 2015 period. Similarly, Boontome et al.^60^ claim that the use of non-renewable energy in Thailand during the 1971–2013 period would have increased CO_2_ emissions. Danish et al.^61^ reached similar results for Pakistan with ARDL and VECM analyses.

**Table 1:** Summary of the selected papers on the energy consumption-CO_2_ emissions relationship.

Author(s)	Country	Period	Methodology	Findings
Soytas and Sarı (2009)	Turkey	1960–2000	VAR, GC	EC⇑ CO_2_⇓
Apergis et al. (2010)	19 developed and developing economies	1984–2007	Panel model	NCEC⇑ CO_2_⇓
Lin and Moubarak (2014)	China	1977–2011	JC, ARDL	REC⇎CO_2_
Jaforullah and King (2015)	USA	1965–2012	JC, VECM, GC	REC⇑ CO_2_⇓
Ben Jebli and Ben Youssef (2015)	Tunisia	1980–2009	ARDL, VECM	REC⇑ CO_2_⇓ NREC⇑ CO_2_⇑
Beşer and Beşer (2017)	Turkey	1960–2015	ARDL	EC⇑ CO_2_⇑
Boontome et al. (2017)	Thailand	1971–2013	Cointegration, causality	NREC⇑ CO_2_⇑
Mirzaei and Bekri (2017)	Iran	2000–2025	Simulation	EC⇑ CO_2_ ⇑
Zoundi (2017)	25 selected African countries	1980–2012	Panel model	REC⇑ CO_2_⇓
Bekhet and Othman (2018)	Malaysia	1971–2015	ARDL, VECM, GC	REC⟺CO_2_
Sinha and Shahbaz (2018)	India	1971–2015	ARDL	REC⇑ CO_2_⇓
Waheed et al. (2018)	Pakistan	1990–2014	ARDL	EC⇑ CO_2_⇓
Belaïd and Zrelli (2019)	9 Mediterranean countries	1980–2014	PMG, ARDL	REC⟺CO_2_
Danish et al. (2018)	Pakistan	1990–2015	ARDL, VECM	EC⇑ CO_2_ ⇑
Chontanawat (2020)	ASEAN	1971–2015	Cointegration, causality	EC⟺CO_2_
Kuşkaya and Bilgili (2020)	USA	1989:1–2017:8	Wavelet analysis	WNEC⇑ CO_2_⇓
Kim et al. (2020)	USA	1973:1–2016:12	ARDL	BEC⇑ CO_2_⇓
Saidi and Omri (2020)	15 major renewable energy-consuming countries	1990–2014	FMOLS, VECM	REC⟺CO_2_
Kuşkaya (2022)	USA	1989:1–2020:1	Wavelet analysis	RSEC⇑ CO_2_⇓
Ozgur et al. (2022)	India	1970–2016	Fourier ARDL	NCEC⇑ CO_2_⇓
Adebayo et al. (2023)	BRICS countries	1990:Q1:2019:Q4	Wavelet analysis	NREC⇑ CO2⇑
Apergis et al. (2023)	Uzbekistan	1985–2020	ARDL	HEC⇑ CO_2_⇓ NREC⇑ CO_2_⇑
Kartal et al. (2023)	France	1970–2021	DYNARDL	NCEC⇑ CO_2_⇓
Kuşkaya et al. (2023)	USA	1990:1–2022:6	Wavelet analysis	TSEC⇑ CO2⇓
Mukhtarov et al. (2023)	Azerbaijan	1993–2019	DOLS	REC⇑ CO2⇓

⟹ (⇏) represent unidirectional (non-) causality. ⟺ (⇎) indicate bidirectional (non-) causality.

*ARDL* auto-regressive distributed lags, *BEC* biomass energy consumption, *CO*_*2*_ carbon dioxide emissions, *DYNARDL* dynamic auto-regressive distributed lags, *DOLS* dynamic ordinary least squares method, *EC* energy consumption, *EG* economic growth, *FMOLS* fully modified ordinary least squares, *GMM* generalized method of moments, *GPM* grey prediction model, *GS2SLS* generalized spatial two-stage least squares, *HEC JC* Johansen cointegration, *LR* long-run, *NCEC* nuclear energy consumption, *NREC* nonrenewable energy consumption, *PMG* pooled mean group, *PVAR* panel vector auto-regression, *REC* renewable energy consumption, *SR* short-run, *TSEC* total solar energy consumption, *VECM* vector error correction model, *WEC* waste energy consumption, *WNEC* wind energy consumption.

The second group of researchers examined the relationship between energy use or consumption (EC) and economic growth (EG). The results were evaluated according to four different hypotheses: growth, conservation, feedback, and neutrality (see Table 2).

- Growth hypothesis (EC⟹EG): it states a unidirectional causality from energy use to economic growth (Narayan and Smyth^62^; Lee and Chang^63^; Apergis and Payne^64^^,^^65^; Bilgili and Ozturk^66^; Magazzino^67^; Ozturk and Bilgili^68^; Aslan^69^; Adams et al.^70^; Ntanos et al.^71^; Luqman et al.^72^; Shahbaz et al.^73^; Gyimah et al.^74^; Espoir et al.^75^; Mohammadi et al.^76^; Simionescu^77^). If the growth hypothesis is valid, policies to reduce energy use have negative effects on economic growth.
- Conservation hypothesis (EG⟹EC): it is valid if there is a unidirectional causality from economic growth to energy usage (Sadorsky^78^; Menyah and Wolde-Rufael^79^; Ocal and Aslan^80^). In this case, policies that change energy usage do not have a negative impact on economic growth.
- Feedback hypothesis (EC⟺EG): it is supported if there is a bidirectional causality between energy use and economic growth (Belloumi^81^; Pao and Fu^82^; Acheampong et al.^7^; Lawal^83^; Simionescu^77^).
- Neutrality hypothesis (EC⇎EG): it implies the absence of any causal relationship between energy use and economic growth (Lee and Chang^63^; Pao and Fu^84^). In this situation, policies changing energy use do not affect economic growth.

**Table 2:** Summary of the selected papers on the economic growth-energy consumption relationship.

Author(s)	Country	Period	Methodology	Supported hypothesis
Lee and Chang (2008)	16 Asian countries	1971–2002	Panel model	Neutrality, growth
Narayan and Smyth (2008)	G7-countries	1972–2002	Panel model	Growth
Belloumi (2009)	Tunisia	1971–2004	VAR	Feedback
Sadorsky (2009)	18 emerging countries	1994–2003	Panel cointegration, FMOLS	Conservation
Menyah and Wolde-Rufael (2010)	USA	1960–2007	VAR	Conservation
Apergis and Payne (2010a)	20 OECD countries	1985–2005	FMOLS	Growth
Apergis and Payne (2010b)	13 Eurasian countries	1992–2007	Panel model	Growth
Ocal and Aslan (2013)	Turkey	1990–2010	Toda-Yamamoto causality	Conservation
Pao and Fu (2013)	Brazil	1980–2010	Cointegration, ECM	Feedback
Bilgili and Ozturk (2015)	G7 countries	1980–2009	Panel model	Growth
Magazzino (2015)	Italy	1970–2009	VAR, VECM	Growth
Ozturk and Bilgili (2015)	51 SSA countries	1980–2009	Panel model	Growth
Pao and Fu (2015)	Mexico	2980–2011	Lotka-Volterra model	Neutrality
Aslan (2016)	USA	1961–2011	ARDL	Growth
Adams et al. (2018)	30 SSA countries	1980–2012	DOLS-FMOLS	Growth
Ntanos et al. (2018)	25 European countries	2007–2016	Panel model	Growth
Luqman et al. (2019)	Pakistan	1990–2016	NARDL	Growth
Shahbaz et al. (2020)	38 REC countries	1990–2018	DOLS, FMOLS	Growth
Acheampong et al. (2021)	23 emerging economies	1970–2015	IV-GMM	Feedback
Gyimah et al. (2022)	Ghana	1990–2015	GC	Growth
Espoir et al. (2023)	African countries	1980–2018	Panel model	Growth
Lawal (2023)	Africa	1980–2019	Frequency domain analysis	Feedback
Mohammadi et al. (2023)	Selected developed and developing countries f	1993–2019	FMOLS Dumitrescu and Hurlin heterogeneous panel causality	Growth
Simionescu (2023)	13 EU countries	2002–2021	Panel model	Feedback, growth

*DOLS* dynamic ordinary least squares; *GC* granger causality; *SSA* Sub-Saharan Africa.

Energy is considered an engine for economic growth. However, energy has undesirable impacts on environmental degradation as it leads to pollutant emissions (Nasir and Rehman^85^). Therefore, the third group of works consists of papers examining the relationship between economic growth and the environment (in particular, CO_2_ emissions). This group presents studies investigating the validity of the Environmental Kuznets Curve (EKC). As shown in Table 3, the validity of the EKC hypothesis has been proven in most of the studies conducted in the environmental-economics literature (Dijkgraaf and Vollebergh^86^; Iwata et al.^87^; Nasir and Rehman^85^; Castiglione et al.^88^; Esteve and Tamarit^89^; Shahbaz et al.^90^; Baek and Kim^91^; Sulaiman et al.^92^; Heidari et al.^93^; Al-Mulali and Ozturk^94^; Bilgili et al.^95^; Dogan and Seker^96^; Ulucak and Bilgili^97^; Koçak and Şarkgüneşi^98^; Köksal et al.^99^; Jahanger et al.^100^; Ozturk et al.^101^; Pata et al.^102^). Contrary to these studies, Roca and Alcántara^103^, He and Richard^104^, Arouri et al.^105^, Ozturk and Al-Mulali^106^, Dogan and Turkekul^107^, Liu et al.^108^ and Pata and Tanriover^109^ do not reach results able to provide empirical support to the EKC hypothesis. Studies investigating the EKC hypothesis for time-series generally adopt ARDL and Johansen Cointegration (JC) methodologies.

**Table 3:** Summary of the selected papers on the economic growth-CO_2_ emissions relationship.

Author(s)	Country(s)	Period	Methodology	EKC hypothesis
Roca and Alcántara (2001)	Spain	1972–1997	Decomposition method	No
Dijkgraaf and Vollebergh (2005)	24 OECD countries	1960–1997	Panel model	Yes
Iwata et al. (2010)	France	1960–2003	ARDL	Yes
He and Richard (2010)	Canada	1948–2004	Cubic parametric models	No
Nasir and Rehman (2011)	Pakistan	1972–2008	JC	Yes
Arouri et al. (2012)	12 MENA countries	1981–2005	Panel cointegration	No
Castiglione et al. (2012)	28 countries	1996–2008	2SLS	Yes
Esteve and Tamarit (2012)	Spain	1857–2007	Threshold cointegration	Yes
Shahbaz et al. (2013)	Romania	1980–2010	ARDL	Yes
Baek and Kim (2013)	Korea	1971–2007	ARDL	Yes
Sulaiman et al. (2013)	Malaysia	1980–2009	ARDL	Yes
Ozturk and Al-Mulali (2015)	Cambodia	1996–2012	GMM,2SLS	No
Al-Mulali and Ozturk (2016)	27 advanced economies	1990–2012	Panel models	Yes
Dogan and Seker (2016)	Top renewable energy countries	1985–2011	Heterogeneous panel with cross-section dependence, cointegration	Yes
Dogan and Turkekul (2016)	USA	1960–2010	ARDL, GC	No
Heidari et al. (2015)	ASEAN countries	1980–2008	Panel regression	Yes
Ulucak and Bilgili (2018)	High, middle, and low-income countries	1961–2013	CUP-FM, CUP-BC	Yes
Bilgili et al. (2016)	17 OECD countries	1977–2010	FMOLS, DOLS	Yes
Liu et al. (2017)	Asian countries	1970–2013	FMOLS, DOLS, OLS	No
Koçak and Şarkgüneşi (2018)	Turkey	1974–2013	Cointegration with structural breaks	Yes
Köksal et al. (2020)	Turkey	1961–2014	JC	Yes
Jahanger et al. (2023)	Top ten manufacturing countries	1990–2020	MMQR	Yes
Jahanger et al. (2023)	Nuclear energy-generating nations	1990–2018	DCCE	Yes
Ozturk et al. (2023)	South Asia	1971–2018	FMOLS, DOLS, PMG	Yes
Pata et al. (2023)	Germany	1974–2018	FADL	Yes
Pata and Tanriover (2023)	Top ten tourism destinations	2004–2018	CUP-FM, CUP-BC, CS-ARDL	No

*CUP-BC* continuously updated-bias corrected, *CUP-FM* continuously updated-fully modified, *DCCE* dynamic common correlated effects, *FADL* fourier auto-regressive distributive lags, *MMQR* method of moment quantile regression.

After reviewing the relevant studies in the literature, one can state that Russia can be considered a reasonable and valuable choice for an applied analysis as it is one of the countries with the highest per capita GHG emissions. Moreover, it is one of the three largest oil-producing countries in the world. On the other hand, this study aims to contribute to the literature in terms of the methodology adopted. In particular, the novelty of this paper is the combination of traditional time-series analyses together with the development of ML techniques.

## Methodology and data

The empirical analysis starts with the inspection of the stationarity properties of the series. We performed several recent unit root and stationarity tests. The Kapetanios and Shin^110^ test is more powerful than the linear unit root tests if there are significant asymmetries. This test is an extended version of the seminal unit root test of Kapetanios et al.^111^. Kapetanios et al.^111^ introduced a model for an Exponential Smooth Transition Autoregressive (ESTAR) process, developing a nonlinear test to allow the structural change to be determined internally. In addition, the Leybourne^112^ Augmented Dickey-Fuller (ADF)-max unit-root test and the Elliott et al.^113^ are performed. Afterwards, the Lo^114^ modified rescaled range test for long-range dependence of a time-series is used, to better understand the features of our data. Then, in order to explore the (eventual) presence of a structural breaks in the dataset, we run the recent Ditzen et al.^115^ test, which implements multiple tests for structural breaks in time-series and panel data models. The number and period of occurrence of structural breaks can be known or unknown. Furthermore, to examine the long-run relationship among the variables, we apply the Bayer and Hanck^116^ combined cointegration approach. This test combines the results of previous cointegration approaches (Engle and Granger^117^; Johansen^118^; Boswijk^119^; Banerjee et al.^120^) and provides Fisher *F* statistics for more conclusive and reliable empirical findings. Finally, this study employs the Breitung and Candelon^121^ Spectral Granger (BCSG) causality test. Such a test is superior to standard causality tests because it can predict the target variables at precise time frequencies. Hence, the technique enables us to identify the historical changes to implement the policy intervention. However, the methodology is limited to a finite time horizon and cannot predict infinite time models.

In recent years, there has been a great interest in causality discovery research and its relevant areas (Spirtes and Zhang^122^; Nogueira et al.^123^). Identifying the individual causal relationships between economic and financial time-series is necessary to characterize the structure of causality in an economic system. Understanding this element is essential in almost all cases involving studying complex economic systems (Zaremba and Aste^124^).

Various techniques have been proposed to investigate causal relationships in time-series. In this work, an approach to detect causality based on GC testing (Granger^125^) is adopted, together with a nonlinear GC test. Thus, it is essential to notice that we are explicitly dealing with time-series, which can show some linear relationships; if these are nonlinear, we must also consider a nonlinear version of the test. Following Granger^125^, Hmamouche^126^, and Gkillas et al.^127^, in order to perform a linear version of the GC test, we can write:

1 $$\begin{array}{c}{Y}_{t}={\alpha }_{0}+\sum_{i=1}^{l} {\alpha }_{i}{Y}_{t-i}+{Z}_{t},\\ \end{array}$$

comparing this model with a second one containing the variable to be tested in the causal relationship (*X*).

2 $${Y}_{t}={\alpha }_{0}+\sum_{i=1}^{l} {\alpha }_{i}{Y}_{t-i}+\sum_{i=1}^{l} {\beta }_{i}{X}_{t-i}+{Z}_{t},$$

where *l* is a parameter representing the lag considered. At the same time, [*α*_*0*_, …, *α*_*l*_] and [*β*_*0*_, …, *β*_*l*_] are the model’s parameters. Finally, the residual term *Z*, is a white noise process. The comparison is performed by considering the predictive power of the causal factor *X* against the information provided only by the lags of the *Y*.

The number of lags is chosen to cover the relevant, informative observations of the past, which may also be pertinent considering the degree of freedom. When the lags are added, the degree of freedom decreases directly with the addition of the lags. Thus, to assess the statistical significance, an *F* test is utilized to examine the validity of the null hypothesis, which states that the series *X* does not Granger cause the series *Y*. The test can be repeated considering both the causality directions (Ye and Zhang^128^).

When the time-series does not change linearly over time, Neural Networks (NNs) are widely used in causality detection (Marcinkevičs and Vogt^129^). So, we considered a nonlinear Granger test based on a NN design. Given a multivariate time-series {*Y*_*1*_, …, *Y*_*p*_} considering both the target variable and also *p* predictors variables, the causality test results considering the Vector Auto-Regressive Neural Network (VARNN) of order *p* model can be interpreted similarly to the classical Granger linear causality test.

Therefore, the VARNN(p) model can be stated as a Multi-Layer Perceptron (MLP) NN model for predicting the behaviour of *Y* over time. According to this approach, in addition to the target variable and its lags, *l* lag values of the variable predictors are considered to make *Y* predictions.

Furthermore, an optimization algorithm based on the stochastic gradient descent algorithm is used, allowing the weights to be updated (Gkillas et al.^127^). The VARNN(p) model can be written in this way:

3 $${Y}_{t}={\phi }_{nn}\left({Y}_{t-1},\dots ,{Y}_{t-l},\dots ,{Y}_{p(t-1)},\dots ,{Y}_{p(t-l)}\right)+{Z}_{t},$$

where ${\phi }_{nn}$ refers to the network function, and *Z*_*t*_ is the residual (Hmamouche^126^). In our case, we have two different models to consider. Model 1 is the following:

4 $${Y}_{t}={\phi }_{1nn}\left({Y}_{t-1},\dots ,{Y}_{t-l}\right)+{Z}_{t},$$

while model 2 can be written as:

5 $${Y}_{t}={\phi }_{2nn}\left({Y}_{t-1},\dots ,{Y}_{t-l},{X}_{t-1},\dots ,{X}_{t-l}\right)+{Z}_{t},$$

Here, the two network functions are represented as ${\phi }_{1nn}$ and ${\phi }_{2nn}$ using the VARNN(p) model. An *F* test is utilized in testing against the null hypothesis of no causality. Again, in this case, this hypothesis is that the economic variable represented by the time-series *X* does not cause the time-series *Y*. If we refuse the null hypothesis, we find evidence of causality.

In order to confirm previous results, we apply an ML unsupervised approach to evaluate the time-series similarity structure. In this way, we adopt a method known as clustering or unsupervised learning to classify data with no prior knowledge of the classes to be categorized (Liao^130^; Aghabozorgi et al.^131^). Therefore, clustering time-series is an unsupervised learning approach that data analysts consider to get some insights into the patterns on a dataset (Ariff et al.^132^; Drago and Talamo^133^).

In general, time-series clustering may be very useful in many applications and scientific areas. In this respect, a growing interest in time-series clustering grows, particularly in the search and analysis for similarities across long-term time-series in fields such as economic or financial applications. For example, time-series clustering can be used for similar group data to be easily analyzed and then used for forecasting purposes (Corona et al.^134^; Franses and Wiemann^135^) or portfolio optimization (Guam and Jiang^136^; Tola et al.^137^).

Clustering can be defined as a technique for classifying objects related to unknown classes. In this respect, time-series can be classified into different groups with no previous information about their participation in a single group (Liao^130^; Wang et al.^138^). On a more technical level, clusters are constructed by grouping statistical observations within a cluster with the most significant similarity between the observations inside the same group and the lowest similarity to those outside the cluster (Fu^139^). However, a typical data clustering approach to time-series depends also on the data structure (homogeneous or not homogeneous time-series, for instance). A different approach to clustering time-series based on temporal representation was proposed by Drago and Scepi^140^ and Drago et al.^141^, which is very suitable for long time-series and high-frequency data.

In this work, we have considered the time-series classified on their structure to identify their similarities in response to shocks. This way, the clustering approach explores and evaluates the data structure to make the econometric analysis more robust. Moreover, the data exploration allows us to evaluate better and interpret the econometric models’ results.

It is possible, for that purpose, to consider an unsupervised ML approach to support the different results of the causality tests. In this respect, we can explore the patterns in the data, expecting that the time-series showing patterns of causality is also characterized by higher similarity. Identifying the level of similarity of the different time-series is possible to imply a measurement of the joint dynamics over time.

Various distances are proposed and used in time-series clustering (Liao^130^; Aghabozorgi et al.^131^; Montero and Vilar^142^; Mori et al.^143^). In order to evaluate the distance between the two-time-series *X*_*T*_ and *Y*_*T*_. We consider four relevant distances in time-series clustering (Leung and James^144^). These distances represent a way to assess the similarity between the different economic time-series considered in our work.

The first distance we use is the Euclidean, derived from the Minkowski distance (Montero and Vilar^142^):

6 $${d}_{\mathrm{Euclidean}}\left({X}_{T},{Y}_{T}\right)={\left(\sum_{t=1}^{T} {\left({X}_{t}-{Y}_{t}\right)}^{2}\right)}^{1/2}.$$

The Euclidean distance is a classical distance used in time-series clustering (Aghabozorgi et al.^131^).

However, a different approach is considered using a second distance, the Frechet one (c and Mannila^145^). The advantage of using this distance is that we cluster the time-series considering the order of observations and the two sets of points in each row.

In this respect, start by considering:

7 $$r=\left(\left({X}_{{a}_{1}},{Y}_{{b}_{1}}\right),\dots ,\left({X}_{{a}_{m}},{Y}_{{b}_{m}}\right)\right),$$

where *a*_*i*_, *b*_*j*_ ∈ {1,…,*T*} and *a*_*1*_ = *b*_*1*_ = 1, *a*_*m*_ = *b*_*m*_ = *T*, where at the same time: *a*_*1*+*1*_ = *a*_*1*_ or also *a*_*1*+*1*_ (and at the same time considering *b* with the same characteristics of *a*) for *i* ∈ {1,…,*m*-1}.

Following Montero and Vilar^142^, Eiter and Mannila^145^, and Driemel et al.^146^, the Frechet distance can be written as:

8 $${D}_{Frechet}\left({X}_{T},{Y}_{T}\right)=\underset{r\in M}{min} \left(\underset{i=1,\dots ,m}{max} \left|{X}_{{a}_{i}}-{Y}_{{b}_{i}}\right|\right).$$

The outliers can affect the Frechet distance (Brankovic et al.^147^), so we consider a third approach: the dynamic time warping distance (Vintsyuk^148^; Hiroaki and Seibi^149^; Giorgino^150^). The dynamic time warping distance, a known approach in many different contexts, was recently considered relevant in economic applications to analyze the joint dynamics between the different time-series (Franses and Wiemann^135^).

Liao^130^ clarified that the advantage of using the Dynamic Time Warping (DTW) distance is that the algorithm can compare discrete sequences with sequences of continuous values where the two series are synchronized within DTW to align them as much as possible. So, the DTW algorithm is a generalization of traditional algorithms used to compare discrete sequences with sequences of continuous values.

As the Frechet distance, we start by minimizing the distance between combinations of observations $({X}_{{a}_{i}},{Y}_{{b}_{i}})$.

Thus, we have:

9 $${D}_{DTW}\left({X}_{T},{Y}_{T}\right)=\underset{r\in M}{min} \left(\sum_{i=1,..,m} \left|{X}_{{a}_{i}}-{Y}_{{b}_{i}}\right|\right).$$

Finally, as the last clustering approach, we consider the Correlation distance. We can follow Golay et al.^151^, Montero and Vilar^142^, Kim and Kim^152^, and Drago and Scozzari^153^. The Pearson’s correlation distance between two time-series can be written as:

10 $${D}_{COR}\left({X}_{T},{Y}_{T}\right)=\sqrt{2(1-\mathrm{COR}(\mathrm{X}, \mathrm{Y}))},$$

where COR is the Pearson’s correlation between the two considered time-series *X*_*T*_ and *Y*_*T*_. The interpretation of the Correlation distance between time-series is essential: when there is a higher correlation between two time-series, their distance becomes closer (Kim and Kim^152^). The Correlation distance is advantageous for capturing and describing a linear pattern between different series.

Nevertheless, it can be demonstrated that the Pearson’s correlation distance on clustering time-series is equivalent to the *z*-score normalized, squared Euclidean distance (Berthold and Höppner^154^). This point allows higher robustness for Pearson’s correlation distance.

This approach evaluates the similarity in the yearly series. The results of the approach can be interpreted as follows: a higher correlation between two time-series exists, and a lower dissimilarity between the two- time-series can be identified simultaneously.

The different distance matrices allow assessing the similarity of each relationship (and also causal relationship) between the time-series, where we explicitly consider simulated time-series in order as a statistical benchmark. Causality relationships, both linear and nonlinear, tend to be characterized by a higher similarity in the time-series dynamics. In economic terms, more substantial similarity is also relevant to identifying some relationships in the data that can be related by other variables acting as common determinants; yet, we expect than a higher similarity is increased by the causality.

**Figure 1:**
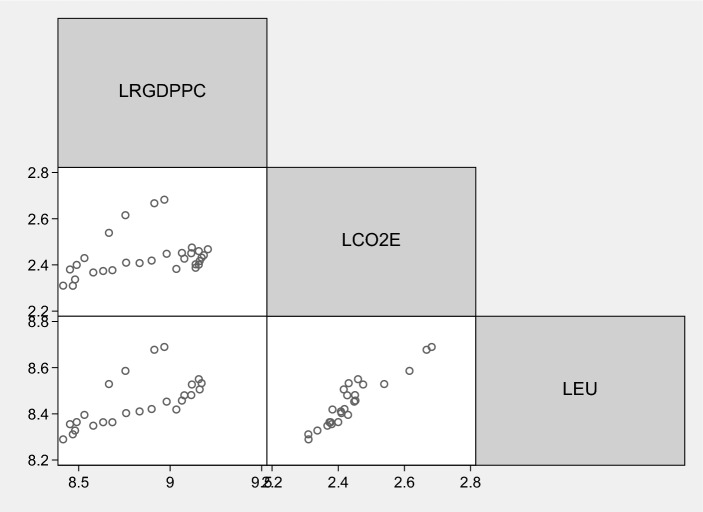
Scatterplot matrices. Sources: authors’ elaborations in STATA.

As concerns our dataset, *CO*_2_ is CO_2_ emissions (in metric tons per capita), from the International Energy Agency (IEA) database (https://www.iea.org/data-and-statistics); *PCGDP* is per capita GDP (in 2000 US$), from the Federal Reserve Economic Data (FRED) database (https://fred.stlouisfed.org/); and *PCEU* is per capita energy use (in kg of oil equivalent), from World Bank (WB) database (https://data.worldbank.org/). To ensure the asymptotic properties, we derived the logarithmic transformations of each variable. The scatterplot matrices of the variables are given in Fig. 1.

## Empirical findings

The results of several unit root and stationarity tests provided in Table 4 show that the analyzed series are non-stationary at levels, since – in general – we cannot reject the null hypothesis (*H*_*0*_) of non-stationarity, for each series.

**Table 4:** Results for unit roots and stationarity tests.

Variable	KSUR	KSSUR	Leybourne	ERS
A: intercept				
LCO2E	− 2.123 (− 2.859)	− 1.674 (− 3.055)	0.656 (− 2.140)	− 2.078 (− 2.608)
LEU	− 1.881 (− 2.964)	− 1.771 (− 3.102)	0.959 (− 1.751)	− 2.655* (− 2.720)
LRGDPPC	− 2.832** (− 2.768)	− 2.543 (− 2.986)	− 1.246 (− 2.329)	− 2.193* (− 2.352)
B: intercept and trend				
LCO2E	− 1.754 (− 3.558)	− 1.695 (− 3.512)	− 0.080 (− 2.969)	− 2.478 (− 3.737)
LEU	− 1.802 (− 3.677)	− 1.774 (− 3.569)	− 0.537 (− 2.258)	− 2.132 (− 3.904)
LRGDPPC	− 2.869 (− 3.446)	− 2.878 (− 3.408)	− 3.337** (− 3.133)	− 3.591* (− 3.619)

*KSUR* Kapetanios and Shin test; *KSSUR*: Kapetanios, Shin, and Snell test; *ERS*: Elliott, Rothenberg, and Stock test. Lag length is chosen through AIC. 5% Critical Values in parentheses. ***p < 0.01, **p < 0.05, *p < 0.10.

To check the previous results, the Lo^114^ Modified Rescaled Range/Standard deviation (R/S) test and the Hurst-Mandelbrot Classical R/S test (Hurst^155^; Mandelbrot^156^) for long-range dependence are performed. The results are given in Table 5.

**Table 5:** R/S test results.

Test statistic	LCO2E	LEU	LRGDPPC
Lo modified R/S	1.82	0.704	3.49
Hurst-Mandelbrot classical R/S	1.64	1.96	2.52

Andrews’s criterion applied. CVs: 90%: [0.861, 1.747]; 95%: [0.809, 1.862]; 99%: [0.721, 2.098].

Applied to our sample, the two R/S tests fail to reject the null hypothesis of no long-range dependence at the 95% significance level for carbon dioxide emissions, whilst the other two series (energy consumption and real GDP) reject the *H*_*0*_ hypothesis. Thus, there is no evidence that the emissions series is long-range dependent, while evidence of long-run dependence in the energy consumption and real GDP emerges.

To analyze the presence of structural breaks in the series the recent Ditzen et al.^115^ test is applied, which implements multiple tests for structural breaks in time-series.

Following Bai and Perron^157^, this test is able to detect multiple breaks at unknown break dates. The results in Table 6 show that, when we test the first hypothesis of no break against the alternative of *s* = 1 break, the null is rejected (at any level). The estimated break points correspond to 1996 (oil crisis) and 2008 (world economic-financial crisis). With the second hypothesis, we test the absence of breaks against a lower and upper limit of breaks (in this case, 1 ≤ *s* ≤ 2); again, *H*_*0*_ is soundly rejected. Now, the estimated break point is 2009. Finally, the last hypothesis tests the null of *s* = 1 break against the alternative of one more break (*s* + 1 = 2); here, we cannot reject the null hypothesis. Thus, we can conclude that a single break appears in these data. To check the robustness of this result, following again the Ditzen et al.^115^ approach, a test for multiple breaks at known break dates has been performed. We get a *W(τ)* test statistic = 13.84 (with a P-Value = 0.00); thus, the previous finding of a break in 2009 is confirmed.

**Table 6:** Results for multiple breaks test.

	Test statistic	1% CV	5% CV	10% CV
supW(τ)^a^	8.65***	6.08	4.88	4.32
UDmax(τ)^b^	13.84***	7.71	5.85	5.08
F(s + 1|s)^c^	0.02	16.84	12.95	11.40

Bai and Perron (1998) ^157^ Critical values. ***p < 0.01, **p < 0.05, *p < 0.10. Trimming: 0.15.

^a^Estimated break points: 1996, 2008, ^b^evaluated at a level of 0.95. Estimated break point: 2009, ^c^*s* = 1.

Since non-stationarity emerges for data at levels, we can test the (eventual) presence of a long-run relationship, or a linear combination of the series which is stationary (having a lower order of integration) (Engle and Granger^117^). As the next step, we check for the (eventual) presence of a long-run relationship among the selected series, applying the Bayer and Hanck^116^ procedure (see Table 7). The first model, which in the deterministic specification does not allow either a constant or a trend, gives a test statistic = 20.0827 (with a 5% Critical value = 10.838 and a 10% Critical Value = 8.457), based on Engle and Granger^117^ and Johansen^158^ tests, and a test statistic = 22.5377 (with a 5% Critical value = 20.776 and a 10% Critical value = 16.171), based on Engle-Granger, Johansen, Boswijk, and Banerjee et al. tests. Looking at these findings, we can assume that a cointegrating relation exists. The second model includes an unrestricted constant, with a test statistic = 14.1139 (with a 5% Critical value = 10.895 and a 10% Critical value = 8.479), based on Engle and Granger and Johansen tests, and a test statistic = 15.9980 (with a 5% Critical value = 21.106 and a 10% Critical value = 16.444), based on all four tests. In this case, we find only a weak empirical sustain in favour of the presence of cointegration. Finally, the last model includes both for a linear and a quadratic trend, with a test statistic = 16.8009 (with a 5% Critical value = 10.858 and a 10% Critical value = 8.451), based on Engle and Granger and Johansen tests, and a test statistic = 32.8171 (with a 5% Critical value = 21.342 and a 10% Critical value = 16.507), based on all tests. Here, the evidence of a cointegrating relationship is very clear, and the null hypothesis can be rejected at any significance level.

**Table 7:** Results for Bayer-Hanck test for cointegration.

Test	Test statistic	P-value
Model 1		
Engle-Granger	− 3.1375*	0.0726
Johansen	29.5223***	0.0006
Banerjee-Dolado-Mestre	0.9454	0.9794
Boswijk	6.1926	0.2992
Model 2		
Engle-Granger	− 1.0758	0.9571
Johansen	32.5652***	0.0009
Banerjee-Dolado-Mestre	− 0.7985	0.9068
Boswijk	7.0497	0.4299
Model 3		
Engle-Granger	− 3.7396	0.1183
Johansen	34.4612***	0.0019
Banerjee-Dolado-Mestre	− 4.1411**	0.0275
Boswijk	21.4575**	0.0121

Model 1: do not include a trend or a constant in the model; Model 2: include an unrestricted constant in the model; Model 3: include a linear trend in the cointegrating equations and a quadratic trend in the undifferenced data. ***p < 0.01, **p < 0.05, *p < 0.10.

**Figure 2:**
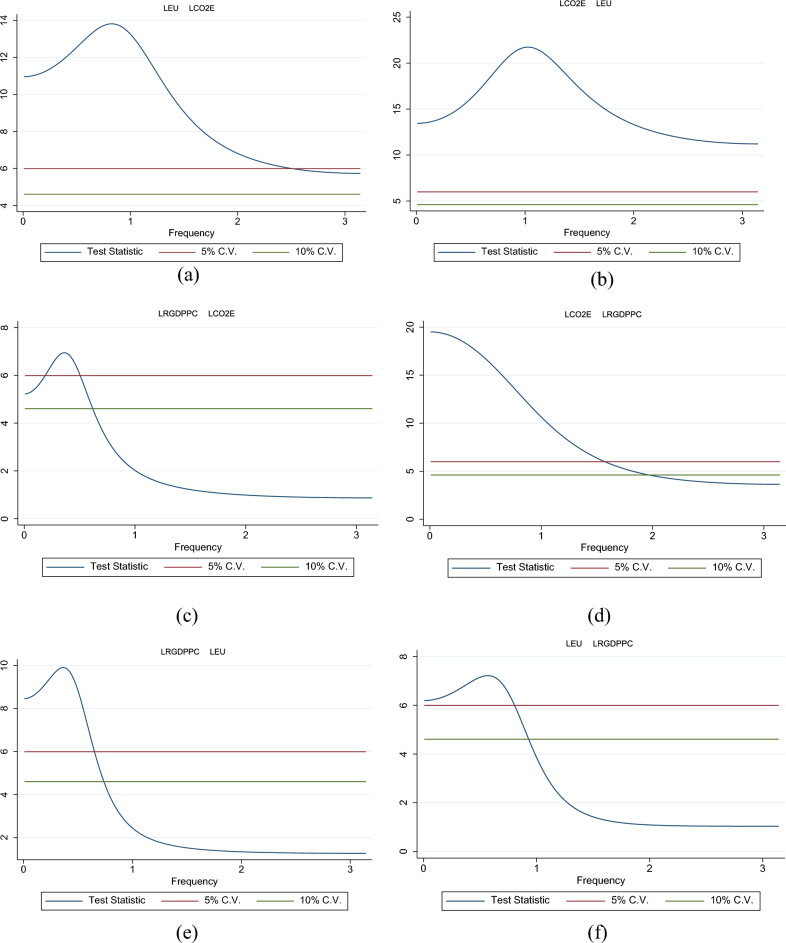
Breitung-Candelon Spectral Granger causality test results. Confidence level on *y-axis*. Geweke-type conditioning was used. The following relationships are empirically tested: LEU → LCO2E: innovation in energy use causes CO_2_ emissions. LCO2E → LEU: innovation in CO_2_ emissions causes energy use. LRGDPPC → LCO2E: innovation in real GDP causes CO_2_ emissions. LCO2E → LRGDPPC: innovation in CO_2_ emissions causes real GDP. LRGDPPC → LEU: innovation in real GDP causes energy use. LEU → LRGDPPC: innovation in energy use causes real GDP. Sources: authors’ elaborations in STATA.

In Fig. 2, the main results of the BCSG test are shown. The relationships among the variables are assessed over the time–frequency domain. Each figure displays the Wald statistics over all frequencies *ω* ∈ (0; *π*). The test statistics for the Granger non-causality from energy use to carbon emissions (Fig. 2a) are significant at the 10% level for all frequencies, while the null hypothesis of no GC is rejected at the 5% significance level for frequency in the range *ω* ∈ (0; 2.50). The causal flow in the reverse direction (from *LCO2E* to *LEU*) shows test statistics above the critical bounds on the whole frequency spectrum (0; 3.14), so that the *H*_*0*_ hypothesis can be easily rejected at any significance level (Fig. 2b). Thus, we can conclude that a bidirectional causal flow – with a feedback mechanism – emerges as regards the energy use-CO_2_ emissions nexus. Real GDP is found to cause emissions at a 5% level in the range *ω* ∈ (0; 0.18) and (0.51; 0.61), and at a 1% level in (0.19; 0.50); while for frequency ≥ 0.62 the null is not rejected (Fig. 2c). On the other hand, CO_2_ emissions cause real GDP at least at a 5% significance level for frequencies ≤ 1.57; at a 10% level in the range *ω* ∈ (1.58; 1.96); then, for *ω* ≥ 1.97 the calculated test statistic is lower than the Critical Values (Fig. 2d). Thus, we found that the causality analyses are really sensitive to the selected frequency. Looking at the entire frequency spectrum, we can state that a unidirectional causal flow running from CO_2_ emissions to real GDP exists. For the last couple of variables, real GDP is found to significantly affect energy use for frequencies ≤ 0.65 at a 5% level in the range; for frequencies in the range *ω* ∈ (0.76; 0.73) at a 10% level in the range; while, for *ω* ≥ 0.74 the calculated test statistic is lower than the Critical Values (Fig. 2e). Instead, energy use Granger causes real GDP up to *ω* = 0.80 at least at a 5% significance level. Therefore, we can state that for low frequencies a bidirectional causal link is discovered; however, for medium and high frequencies, the empirical evidence is in favor of the neutrality hypothesis.

Finally, generally speaking, the test results according to Hosoya-type conditioning are qualitatively similar.

In addition, as robustness checks, we implemented unsupervised ML techniques on clusterization. The computational approach has been developed in R programming language. First, the three variables are transformed, deriving their log transformations first differences. Then, we perform the linear GC test. Finally, a nonlinear causality test is conducted.

The results for the linear Granger tests are given in Table 8. It emerges a bidirectional causal flow (feedback mechanism) between energy use and CO_2_ emissions. Thus, energy use causes CO_2_ emission, implying that an increase in energy use could lead to an increase in CO_2_ emissions, and vice versa. These results imply that Russia was dependent on carbon energy use for its fast economic growth in the past, causing considerable CO_2_ emissions. Furthermore, the “neutrality hypothesis” holds regarding the relationship between energy use and aggregate income, which states that there is no causality (in either direction) between the variables. Under the “neutrality hypothesis”, the energy conservation measures do not adversely affect the economic growth path. Therefore, being these two variables mutually independent, we can state that energy consumption is not related to GDP, so that neither conservative nor expansive energy policies may have an effect on economic growth. Finally, a weak causality running from carbon emissions to real GDP is found (which is significant at a 10% level).

**Table 8:** Linear Granger causality tests results.

	LEU → LCO2E	LCO2E → LEU	LCO2E → LRGDPPC	LRGDPPC → LCO2E	LEU → LRGDPPC	LRGDPPC → LEU
Lag parameter	3	3	3	3	3	3
Granger causality index	0.7502	0.8881	0.4593	0.2716	0.2412	0.1721
F test	5.2142** (0.0126)	6.6762*** (0.0050)	2.7205* (0.0841)	1.4563 (0.2690)	1.2732 (0.3218)	0.8762 (0.4769)
5% CVs	3.411	3.411	3.411	3.411	3.411	3.411

P-values in parentheses. ***p < 0.01, **p < 0.05, *p < 0.10.

Table 8 also reports the Granger Causality Index to quantitatively evaluate the causality nexus. Here, it is observed that the higher scores are reached for the direction of causality running from energy use to CO_2_ emissions (0.75), from CO_2_ emissions to energy use (0.89), and from CO_2_ emissions to aggregate income (0.46).

In addition, the nonlinear GC tests are performed. The findings in Table 9 clearly clarify that none of the tested relations has a nonlinear feature. Indeed, we cannot reject the null hypothesis for each test.

**Table 9:** Nonlinear Granger causality tests results.

	LEU → LCO2E	LCO2E → LEU	LCO2E → LRGDPPC	LRGDPPC → LCO2E	LEU → LRGDPPC	LRGDPPC → LEU
Lag parameter	3	3	3	3	3	3
Granger causality index	0.0201	0.0776	0.1422	0.0748	0.1163	0.0915
F test	0.0135 (0.9999)	0.0538 (0.9984)	0.1019 (0.9916)	0.0518 (0.9986)	0.0822 (0.9951)	0.0638 (0.9975)
5% CVs	6.163	6.163	6.163	6.163	6.163	6.163

P-values in parentheses. ***p < 0.01, **p < 0.05, *p < 0.10.

The relationship between changes in CO_2_ emissions and changes in energy use are well-established in the literature internationally. We tested this causal relationship for Russia, finding that they are consistent with a lot of previous studies. The relationship among energy use, CO_2_ emissions, and real GDP has been empirically examined for a large variety of countries (Sohag et al.^159^; Li and Su^160^; He et al.^161^). This literature showed that CO_2_ emissions increase when energy use increases, mainly from fossil fuels. In the same way, a decrease in energy use can lower CO_2_ emissions, positively affecting the environment. Energy production from fossil fuels is the primary source of CO_2_ emissions worldwide. Therefore, considering the role of the population and the role of urbanization as well, energy use and CO_2_ emissions are positively correlated (Shi^162^; Poumanyvong and Kaneko^163^). However, not all changes in energy use will have the same impact on CO_2_ emissions. For example, if there is a shift from using fossil fuels to renewable energy sources, this may cause a CO_2_ emissions reduction although in the presence of an energy use raise (Hickman et al.^164^). On the other hand, efforts to reduce energy consumption and transition to renewable energy sources can diminish CO_2_ emissions (Li and Su^160^; Salahuddin^165^). However, a variation in CO_2_ emissions due to energy consumption can lead to a fluctuation in energy use in the medium-long term (Hwang and Yoo^166^). Similar considerations can be made considering the role of GDP. In fact, a CO_2_ increase can follow the economic growth process (Zhang et al.^167^). On the other hand, shocks to CO_2_ series can determine the real GDP (Bozkurt and Akan^168^; Saidi and Hammami^169^).

Overall, the relevance of the results is twofold. Firstly, it is possible to observe an abundant literature on the bidirectional causal flow between the energy use and CO_2_ emissions. This is very important because investigating the causality of the relationship is more straightforward in determining policy implications and measures. Nevertheless, our causality analyses raised a relevant point: causality tests are sensitive to the frequency domain. At the same time, it is worth noting that we considered Russia as case study, which is more relevant than ever and can have critical international implications.

As a final robustness check, let us consider four different clustering methodologies to evaluate the similarity of the time-series induced by the causality. First, it is possible to analyze if the time-series tend to be very similar and affected by common economic shocks. In the case of causality, we can explicitly identify a strong relationship between the different time-series involved. Thus, in this case, we expect to observe a more substantial similarity between the series (see Table 10).

**Table 10:** Time-series clustering results.

	LCO2E	LRGDPPC	LEU
Euclidean distance			
LRGDPPC	0.2345		
LEU	0.0717	0.2101	
STS	1.5102	1.5944	1.5164
Frechet distance			
LRGDPPC	0.0914		
LEU	0.0385	0.0787	
STS	1.0657	1.1064	1.0640
DWT distance			
LRGDPPC	0.8440		
LEU	0.4769	0.7465	
STS	4.5504	5.2890	4.5202
Correlation distance			
LRGDPPC	0.6183		
LEU	0.3159	0.5302	
STS	1.7857	1.8916	1.8354

We simulate an AR(1) process, using this statistical benchmark to evaluate the similarity between the real series and the simulated one. First, the simulated series is constructed considering the AR(1) parameter = 0.7 and subsequently transformed in the same way as the original series (taking the logarithms and the first difference). On the basis of causality findings, a more substantial similarity between energy use and carbon emissions and a weaker one between CO_2_ emissions and GDP is expected. At the same time, we do not expect any similarity between the simulated time-series and the others.

A more remarkable similarity between emissions and energy consumption emerges, with a lower similarity for emissions and GDP. Finally, we found only low similarity scores between the three original variables and the simulated series. Therefore, these results soundly confirm our previous findings.

Applying the Correlation distance to the data we observe the highest similarity between energy use and carbon emissions (a lower dissimilarity expressed by the distance). This result, again, confirms the previous ones. Therefore, it is possible to affirm that the same standard temporal shocks can simultaneously fuel highly similar short-run dynamics for these series.

We can postulate the existence of a cluster of time-series that is determined by a stronger relationship between energy use and emissions because of the stronger correlations. The lower dissimilarity is reasonable given that they are simultaneously causing each other, while a weaker relationship between emissions and GDP exists.

Because of the exploratory nature of the analysis, however, the method does not formally identify the cluster. In any case, the ML analysis confirms and robustifies the time-series findings.

## Conclusions and policy implications

In 2017, the Paris Climate Agreement came into force. It is aimed at implementing the United Nations Framework Convention on Climate Change and at maintaining an average global temperature growth below 2 °C. Although the agreement does not contain specific obligations for countries on GHG since the beginning of its implementation, many studies have recorded the stabilization of global CO_2_ emissions deriving from the combustion of fossil fuels and industrial processes. The main practical conclusion of these studies is that stabilization and even the reduction of GHG emissions are possible without damaging economic growth. Therefore, in the current study we inspected the link among CO_2_ emissions, energy use, and economic growth in Russia. In particular, the novelty of this paper is the combination of traditional time-series analyses together with the development of a new ML model. It is an important experiment because, to the best of our knowledge, in the related literature no ML experiments have been conducted for the Russian case. The econometric model appears with a test that anticipates the ML model. There is a direct relationship among the selected variables. The primary variable, in this case, is economic growth, which leads to a substantial increase in energy consumption, hence an increase of CO_2_ emissions in the atmosphere. However, due to the hazardous impacts of CO_2_ emissions, the strict regulations which promote the use of clean energy as opposed to fossil fuel, economic growth, and energy consumption do not seem to have a direct impact on the amount of CO_2_ emissions levels. In this case, economic growth increases the consumption of energy, and therefore, the country should concentrate on producing and consuming green energy. The policy would make it easier to control carbon emissions, hence conserving the environment. In this paper, therefore, we have studied the relationship between carbon dioxide emissions, economic growth, and energy use through two different approaches. In the qualitative one, there emerged the existence of a direct relationship with energy consumption policies and carbon dioxide emission policies that regulate gas consumption and emissions. Besides, we have seen how economic growth and CO_2_ emissions are linked to the use of coal for energy. In fact, lower economic growth could encourage the use of easily accessible and low-cost resources.

On the contrary, continuous economic growth reduces the overall CO_2_ emissions in terms of quality. We obtained the same results through quantitative analysis. In particular, after conducting numerous stationary tests, we have analyzed in detail the decomposition of generalized variance, and we can see a long-term relationship between our checks. However, a shock to GDP per capita affects both CO_2_ emissions and energy use for some periods. This result confirms the qualitative analysis suggesting long-term structural investments capable of replacing conventional energy sources with alternative ones. In this way, CO_2_ emissions will be reduced even in the presence of hypothetical shocks.

The policy implications of our analysis can be derived by combining the empirical results obtained with the analysis of emission reduction policies already initiated in Russia and, finally, the current geopolitical context.

With regard to the empirical results, in a nutshell, the work revealed a bidirectional causal flow between energy consumption and CO_2_ emissions and the predominance of the “neutrality hypothesis” for the relationship between energy consumption and GDP. Strictly speaking, this means that in Russia the most exploited energy sources are fossil fuels: when energy consumption grows, supply meets this demand by increasing production from mainly fossil fuels; however, the relationship between energy consumption and GDP growth is not strictly relevant, so that GDP growth does not strictly depend on the increase in energy consumption.

From an environmental perspective, despite the dominance of fossil fuels in the Russian economy, since 2009 there has been an interesting debate in the country on the regulation of greenhouse gas emissions. Indeed, in 2009 the Climate Doctrine of the Russian Federation was adopted (Climate Doctrine of the Russian Federation^170^), then between 2008 and 2012 Russia complied with the first commitment of the Kyoto Protocol, which obliged it not to increase the volume of greenhouse gas emissions compared to the base year 1990 (Andonova^171^). Finally, in 2019, Russia joined the Paris Agreement and subsequently submitted its Nationally Determined Contributions (NDCs), which set a 2030 emissions target of 70% of 1990 emissions. This target is unambitious, in fact it technically implies an increase in emissions compared to current levels: in 1990–2018, Russian GHG emissions decreased by 30.4% (47.6% if emissions from land use, land use change and forestry are included), so if emissions from land use are excluded from the calculation, the 2030 target has already been reached; if they are included in 2030, emissions could increase by 27.3% compared to 2018 levels (Stepanov and Makarov^172^).

The final aspect to analyses is the geopolitical context. The Ukrainian-Russian conflict has created certain contextual conditions that make the future of decarbonization in Russia very complex. In particular, the restriction of trade relations between the EU and Russia has, in our view, two important implications: (1) Europe has accelerated its decarbonization process with the main objective of freeing itself from its energy dependence on Russian fossil sources, which implies a lower availability of financial resources related to the decrease in gas exports and a greater availability of fossil sources to accompany domestic industrialization processes; (2) Russia now has very limited access to import advanced technologies in the field of renewables and clean energy, technologies that are indispensable to advance decarbonization processes.

Taken together, these considerations lead to the outline of a specific policy path for Russia, which could include a mix of infrastructure upgrades and energy efficiency policies. Moreover, the levelized cost of energy (LCOE) of solar and wind is higher than that of coal in Russia, effectively requiring large subsidies to promote these new sources. Only nuclear power has an LCOE in line with coal. It should also be noted that the non-electrified areas of the country are the most remote. In these areas there is a widespread presence of traditional generators that require hydrocarbons, the transport costs of which are very high, and consequently the LCOE of these areas is very high (Dolgushin et al.^173^^,^^174^). It is precisely these areas that could be targeted by an intensive plan of electricity infrastructure development to reduce reliance on outdated and polluting technologies such as traditional generators, accompanied by a significant development of the electricity generation system based on nuclear and CCGT (Combined Cycle Gas Turbines), technologies that do not need to be imported. The combination of these policies could, according to our empirical analysis, lead to an increase in energy intensity with a corresponding increase in emissions. Therefore, in parallel with the empirically tested dominance of the “neutrality hypothesis” for the relationship between energy consumption and GDP, Russia should develop an energy efficiency policy involving the country’s main industrial centers, again with the dual aim of improving the environmental impact of energy end-uses and promoting a national industrial chain in this sector.

The challenge of decarbonizing Russia is complicated by two structural aspects of the country’s energy system: the lack of convenience in adopting RES (Renewable Energy Sources) technologies such as solar and wind power; and the lack of a national electricity grid (about two-thirds of the Russian Federation’s territory, with just over 20 million inhabitants, is not electrified).

On the other hand, it should be noted that the country’s non-electrified areas are the most peripheral. In these areas, the presence of traditional generators that require hydrocarbons, whose transport costs are very high, is widespread.

Furthermore, the challenge for Russia in the coming years is to develop a new strategy for the development of its energy sector, which enters a zone of high turbulence – even in the absence of the influence of the climate change agenda – due to increasing global competition, growing technological isolation, and financial constraints (Mitrova and Melnikov^174^).

Finally, considering the current conflict between Russia and Ukraine, the rise in the price of natural resources has a potential domino effect on the world economy. The shortage of commodities, in fact, pushes up consumer prices, that is, inflation. To stem the increase in the cost of living, central banks are then forced to raise interest rates, thus reducing the amount of liquid money in circulation. Yet, the increase in interest rates usually also causes a slowdown in consumption expenditures, private investments, and the general economy, with negative effects on employment, which is always closely linked to the dynamics of GDP. In addition, when central banks raise interest rates, there is also an appreciation of the national currency as international investors tend to buy financial assets in those countries that offer higher interest on their government debt. This measure implies two negative effects: the increase of the imports (which causes a deterioration of the balance of payment equilibrium), and tensions on national public accounts, which may provoke a financial insolvency.

Finally, the empirical results obtained were analyzed in the light of the decarbonization policy already initiated by Russia and the current economic and political context. The resulting policy implications suggest that Russia’s decarbonization process could be continued through infrastructural investments in the transmission network to reduce the dependence of the country's peripheral areas on the outdated and polluting generation technologies used today, with the associated expansion of nuclear and CCGT generation capacity. At the same time, emissions could be reduced through energy efficiency processes.

## Supplementary Information

Supplementary Information.

## Data Availability

Data are available upon reasonable request to the Corresponding Author. However, raw data are also attached as Supplementary Material.
